# Detection of infections with hepatitis B virus, hepatitis C virus, and human immunodeficiency virus by analyses of dried blood spots - performance characteristics of the ARCHITECT system and two commercial assays for nucleic acid amplification

**DOI:** 10.1186/1743-422X-10-72

**Published:** 2013-03-05

**Authors:** R Stefan Ross, Oumaima Stambouli, Nico Grüner, Ulrich Marcus, Wei Cai, Weidong Zhang, Ruth Zimmermann, Michael Roggendorf

**Affiliations:** 1 Institute of Virology, National Reference Centre for Hepatitis C, University Hospital Essen, University of Duisburg-Essen, Essen, Germany; 2 Department of Infectious Disease Epidemiology, Robert Koch Institute, Berlin, Germany

**Keywords:** Hepatitis B virus infection, Hepatitis C virus infection, Human immunodeficiency virus infection, Dried blood spots, Intravenous drug users, Serological testing, Molecular testing, Real-time PCR, Transcription-mediated amplification

## Abstract

**Background:**

Nowadays, dried blood spots (DBS) are primarily used to obtain diagnostic access to risk collectives such as intravenous drug users, who are prone to infections with hepatitis B virus (HBV), hepatitis C virus (HCV), and human immunodeficiency virus (HIV). Before DBS analyses can be used in this diagnostic context, however, a comprehensive evaluation of its performance characteristics must be conducted. To the best of our knowledge, the current study presents for the first time such essential data for the Abbott ARCHITECT system, which is currently the worldwide leading platform in this field of infection diagnostics.

**Methods:**

The investigation comprised 1,762 paired serum/DBS samples and a total of 3,524 determinations with the Abbott ARCHITECT HBsAg, anti-HBc, anti-HBs, anti-HCV and HIV-1-p24-antigen/anti-HIV 1/2 assays as well as with the artus HBV LC PCR and VERSANT HCV RNA qualitative (TMA) tests.

**Results:**

In the context of DBS testing, a specificity of 100% was recorded for the seven serological and molecular biological assays. The analytical sensitivity of HBsAg, anti-HBc, anti-HBs, anti-HCV, HIV-1-p24-antigen/anti-HIV 1/2, HBV DNA, and HCV RNA detections in DBS eluates was 98.6%, 97.1%, 97.5%, 97.8%, 100%, 93%, and 100%, respectively.

**Discussion/conclusions:**

The results obtained indicate that it is today possible to reliably detect HBsAg, anti-HBc, anti-HBs, anti-HCV and HIV-1-p24 antigen/anti-HIV 1/2 with state-of-the-art analytical systems such as the Abbott ARCHITECT in DBS eluates even when a comparatively high elution volume of 1,000 μl is used. They also provide evidence for the inherent analytical limits of DBS testing, which primarily concern the anti-HBc/anti-HBs system for individuals with HIV infections and nucleic acid tests with relatively low analytical sensitivity.

## Background

Blood samples dried on filter paper (dried blood spots, DBS) were first used in human medical diagnostics by Guthrie and Susi as a simple test for detecting phenylketonuria [1]. Taking capillary blood samples is much easier than a venipuncture and does not require prompt removal of the cellular components. Furthermore, antibodies, many medications and their metabolites as well as nucleic acids remain stable for much longer periods in DBS than in whole blood, plasma or serum. Thus, it is not surprising that the application field of DBS testing has steadily broadened in the last five decades [2]. Today, DBS analysis is predominantly used in the course of the diagnostics and therapy monitoring of chronic diseases, such as infections caused by hepatitis B virus (HBV), hepatitis C virus (HCV) and human immunodeficiency virus (HIV) in those parts of the world in which cost-intensive, on-site laboratory medical infrastructure cannot be made available for economic reasons [3-6]. In high income countries, the “filter card technique” is primarily employed to facilitate diagnosis of viral infections in populations who have only limited access to healthcare. Intravenous drug users are a large fraction of these groups of people. They currently represent the main driving force in HCV epidemiology in industrialized countries and generally benefit from the use of DBS analysis with regard to their diagnostic detection [2,7-13].

The use of DBS thus offers many advantages in the pre-analytical phase. However, from a purely laboratory medical perspective, it is to be viewed critically because it not only requires a time-consuming, manual sample preparation, but also functions only with haemolytic eluates, which are considered to be a problematical test material per se [14]. There exist neither generally accepted protocols for drying and elution of DBS [2,4,15], nor is it known whether commercially available procedures for serological or molecular HBV, HCV and HIV tests react more or less similarly to the selected DBS elution conditions. Consequently, comprehensive evaluations have to be performed during the preliminary phases of any study that desires to use the DBS technique [2].

To the best of our knowledge, the following study for the first time presents data regarding the analytical performance characteristics of testing HBsAg, anti-HBc, anti-HBs, anti-HCV and HIV-1-p24-antigen/anti-HIV 1/2 in DBS eluates with the Abbott ARCHITECT system, i.e. the currently most frequently used platform worldwide in this field of serological diagnostics (Abbott Diagnostics, personal communication). For the detection of HBV DNA and HCV RNA from serum and DBS eluates, a real-time PCR or an isothermal amplification technique were applied. Since the DBS testing is to be subsequently employed in a multicentric, cross-sectional study entitled “Drugs and Chronic Infectious Diseases”, for which a relatively high sample throughput in a comparatively short time is expected, the whole analytical evaluation had to be based on elution conditions suitable for routine use and was not designed for their optimisation for every individual parameter.

## Results

The analytical tests comprised 1,762 paired serum/DBS samples. They originated from 726 patients and formed the basis for a total of 3,524 determinations. The results of the comparative serum/DBS eluate investigations are summarised in Table 1.

**Table 1:** Analytical specificity and sensitivity of the DBS eluate testing for markers of HBV, HCV and HIV infections compared to serum analyses

**Parameters**	**Serum**		**DBS eluates**		**Specificity (%)/ 95% CI**^**a)**^	**Sensitivity (%)/ 95% CI**^**a)**^
	**n. d. (N)**	**pos. (N)**	**n. d. (N)**	**pos. (N)**		
**HBV**						
HBsAg	159	140	161	138	100/97.7 – 100	98.6/94.9 – 99.8
Anti-HBc	101	204	129^b)^	176^b)^	100/97.2 – 100	86.3/83.0 – 91.8^b)^
			107^c)^	198^c)^		97.1/93.9 – 99.0^c)^
Anti-HBs	153	157	162^b)^	148^b)^	100/97.6 – 100	94.3/90.0 – 97.5^b)^
			157^c)^	153^c)^		97.5/93.8 – 99.3^c)^
HBV DNA	50	100	107	93	100/96.1 – 100	93.0/92.9 – 93.1
**HCV**						
Anti-HCV	160	179	164	175	100/97.7 – 100	97.8/96.0 – 100
HCV RNA	50	100	50	100	100/96.1 – 100	100/98.0 – 100
**HIV**						
HIV-1-p24/anti-HIV 1/2	97	112	97	112	100/96.3 – 100	100/96.8 – 100

The results of the serum analyses served as reference for the calculation of the analytical performance characteristics of the DBS testing. — n. d.: not detectable; pos.: positive.

^a)^ 95% confidence interval calculated according to Clopper and Paerson [50]. ^b)^ Calculated with inclusion of the results obtained from HIV infected individuals. ^c)^ Calculated without the results recorded from HIV infected individuals.

### HBV detections

**Figure 1:**
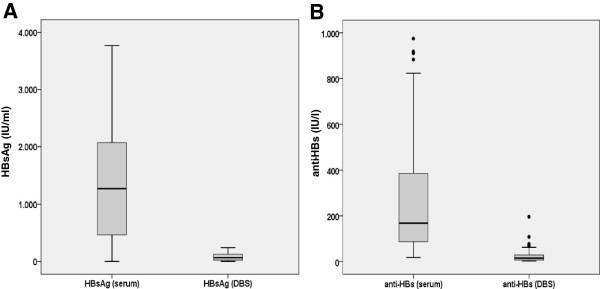
**Distribution of the HBsAg (A) and anti-HBs concentrations (B) in 45 or 107 serum/DBS pairs, respectively, for which titrated values were available.** The “whiskers” on the box plots each represent 1.5 times the interquartile range. Values that lie above or below these thresholds appear separately and are thus tagged as outliers.

A total of 299 serum/DBS samples were tested for HBsAg. One hundred fifty-nine sera were HBsAg negative and 140 HBsAg positive. In 45 paired analyses - for each of which titrated HBsAg concentrations were available - averages of 1,321 IU/ml (serum) and 83 IU/ml (DBS eluates) were recorded (Figure 1). Overall, two discrepant HBsAg results were found. The corresponding test materials were obtained from two patients with chronic HBV infections under antiviral treatment; one of whom was anti-HIV positive. The HBsAg concentrations in the two sera were 749 IU/ml and 6 IU/ml. Thus, a specificity of 100% and a sensitivity of 98.6% were calculated for the HBsAg determination from DBS.

The anti-HBc test was evaluated for a total of 305 paired serum/DBS samples. Overall, 101 of these specimens exhibited no anti-HBc in the serum or in the whole-blood eluates. Twenty-eight patients with positive anti-HBc in serum were not detected by the DBS testing. Twenty-two of these people had suffered from a HBV infection; six exhibited the serological finding “anti-HBc alone”. Twenty-two of the 28 affected individuals were infected with HIV. Of the 22 resolved HBV infections that were not detected in the DSB analysis, 20 were assessed as “anti-HBs positive alone” and thus were falsely classified as “condition after vaccination”. On the basis of all 305 paired anti-HBc determinations, a specificity of 100% and a sensitivity of 86.3% resulted for the testing of the whole blood eluates. In contrast, if subjects with HIV infections had been excluded, a sensitivity of 97.1% was determined.

The analysis of the anti-HBs determinations comprised 310 serum/DBS samples. For specimens reactive in both test materials, 107 titrated anti-HBs concentrations were available; on average they exhibited concentrations of 280 IU/l (serum) and 23 IU/l (DBS eluate) (Figure 1). In total, nine discrepant results occurred. In all these cases, serum anti-HBs concentrations of 11 to 26 IU/l, which were too low to be detected after elution of the dried whole blood, were found. The uncorrected specificity and sensitivity of the anti-HBs testing from DBS thus amounted to values of 100% and 94.3%, respectively. If the samples that originated from HIV positive persons were not included, the sensitivity rose to 97.5%.

DBS testing of 50 individuals negative for HBV DNA did not reveal any non-specificity. The determination of the sensitivity of the qualitative HBV DNA testing from whole blood eluates was performed on 100 specimens (mean DNA concentration: 1,573,898 IU/ml, range: < 357 - > 17,860,000 IU/ml) and yielded a value of 93.0%. Thus, seven samples with low serum HBV DNA concentrations between 409 and 3,643 IU/ml were not detected by the DBS testing.

### HCV detections

Anti-HCV antibodies were determined in 339 whole blood samples as well as in the sera obtained from them. The measurements of the DBS eluates achieved a specificity of 100% and a sensitivity of 97.8%. The four false negative anti-HCV results all involved patients whose sera showed weak anti-HCV reactivities in immunoassay (S/Co 1.2 – 2.65). Only in one case could the positive reaction for anti-HCV also be confirmed in immunoblot. As, in addition, no HCV RNA was detectable for any of those individuals affected (in some cases repeatedly), it is very probable that HCV infections, from which the persons had long since recovered, had been present and could not be detected by DBS testing because of the very low antibody concentration.

DBS testing of 50 HCV RNA-negative individuals did not result in any non-specificity. Of 100 sera which had proven to be HCV RNA positive (mean HCV RNA concentration: 1,415,944 IU/ml, range: 2,479 - >7,692,000 IU/ml), corresponding whole blood aliquots were available. The comparative investigation resulted in an analytical sensitivity of 100% for HCV RNA determinations from DBS eluates.

### HIV detections

The analysis of 209 serum/DBS eluate pairs for HIV-1-p24-antigen/anti-HIV 1/2 provided consistently corresponding results and thus a specificity and sensitivity of 100% for the DBS testing in each case.

## Discussion

To the best of our knowledge, the current study for the first time presents data on the analytical performance characteristics of testing HBsAg, anti-HBc, anti-HBs, anti-HCV and HIV-1-p24-antigen/anti-HIV 1/2 in DBS eluates with the Abbott ARCHITECT system, i.e. the currently most frequently used platform worldwide in this field of serological diagnostics (Abbott Diagnostics, personal communication). Our whole analytical evaluation had to be based on elution conditions suitable for routine use and was not designed to achieve an optimisation for every individual parameter. Consequently, the DBS for the five antigen and antibody tests were eluted with a total of 1,000 μl of a PBS-based buffer with Tween 20 overnight at room temperature [16]. The whole blood eluates for the performance of HBV DNA and HCV RNA tests were prepared under identical conditions in a second operation. The analytical tests comprised 1,762 paired serum/whole blood samples. They originated from 726 patients and were the basis for a total of 3,524 determinations.

There was no analytical non-specificity in serological and molecular biological DBS testing.

The detection of HBsAg positive materials from whole blood eluates succeeded with a sensitivity of 98.6% to a similarly high degree as in previous studies, which had in part used a much smaller elution volume of, for example, 100 μl [17], 250 or 600 μl [18], or 500 μl [19]. In the two non-detected DBS samples, there were HBsAg concentrations of 749 IU/ml and 6 IU/ml. Such a low antigenaemia would probably hardly ever occur in currently injecting drug users chronically infected with HBV because the majority of those people does not receive antiviral treatment [20].

Whether the serum anti-HBc/anti-HBs system for the differentiation of previous HBV infections, on the one hand, and antibody titres as a result of vaccination, on the other hand, is generally also appropriate in DBS eluates has been very rarely investigated to date. Komas and co-workers [19] determined an analytical specificity and sensitivity of 100% for both tests in 15 and 10 corresponding serum/DBS pairs, respectively. In contrast, Tappin et al. [21] were only able to detect anti-HBc antibodies in DBS eluates from 7-day old neonates in 44 of 56 cases (sensitivity: 79%) with a modified hemagglutination assay. Another investigation indicated in an exemplary manner that the anti-HBc/anti-HBs system definitely reacts critically to dilution [18]. Whereas the anti-HBc antibodies in the whole blood eluates were detected independently of the elution volumes (150 or 600 μl) in twelve patients chronically infected with HBV, twelve resolved HBV infections could only then be correctly recorded if the quantity of buffer used to eluate the DBS did not exceed 250 μl. The observations we made on the Abbott ARCHITECT system with an elution volume of 1.000 μl confirm these results communicated by Villa et al. [18] in as much as the anti-HBc/anti-HBs system completely failed when it was used on individuals infected with HIV. In addition, our results prove that immunocompetent persons can also be tested false negative for anti-HBc and anti-HBs in rare cases. This applies particularly to subjects with very low anti-HBc and anti-HBs concentrations in serum.

As a result of its comparatively high detection limit of 100 IU HBV DNA/ml, the artus HBV LC PCR Kit [22] used for DBS testing in this study turned out to be just as reliable as the Cobas Amplicor HBV Monitor Test [23,24] or an in-house rt-PCR [25] and was therefore not superior to older procedures with conventional endpoint detection [26]. For the projected study “Drugs and Chronic Infectious Diseases”, in the course of which DBS testing is to occur, this means that the presumably small number of study participants who had received antiviral treatment could be overlooked, just as could those with a chronic HBV infection and a generally low viremia on the basis of the serological finding “anti-HBc alone” [27].

The detection of anti-HCV antibodies with the ARCHITECT system was a predominantly smooth process for whole blood eluates. The sensitivity of 97.8% determined in the investigation of 179 serum/DBS pairs corresponded to the results obtained in existing studies [16,28-31], which however had worked with an elution volume that was lower by a factor of 5 to 10. In addition, the protocols for anti-HCV detection had been appropriately optimised, in contrast to our analyses, by, e. g., stipulating their own cut-off points [16,29,31] or by increasing the sample volumes used from 20 μl to 100 μl [31]. The fact that in our study four patients with presumably already long since resolved HCV infections were not recognised clearly illustrates the limits of the DBS technique and this fact should be carefully considered when employing this approach in collectives such as intravenous drug users. In this context, due to the high anti-HCV prevalence, a substantial absolute number of people with resolved HCV infections and consequently low anti-HCV concentrations are to be expected [32]; they could, at least partially, evade detection by DBS testing due to the dilution used.

The excellent analytical sensitivity of the HCV RNA test with TMA (circa 5 IU HCV RNA/ml) [33,34] is also reflected in the results of our DBS analysis and allows this approach to appear more promising than the use of an in-house real-time PCR procedure [35] or a multiplex methodology with detection of the amplification products by means of SYBR Green [36]. However, by way of qualification, it must be said that the mean serum HCV RNA concentration in the 100 samples that we examined with approximately 1.4 million IU/ml was relatively high. Since a dilution factor of 16.5 – 18.2 between HCV RNA positive sera and whole blood eluates obtained with a volume of 500 μl was calculated for determinations with TMA in a previous investigation [37], an approximate TMA detection limit of 165 – 182 IU HCV RNA/ml may be assumed under the conditions that we used (1,000 μl elution volume) for the DBS testing. Therefore, the two samples with HCV RNA concentrations of 178 and 331 IU/ml, which Tuailon et al. [31] classified as false negative, could possibly also have escaped from our TMA analysis. This means that a difference in sensitivity of 97% [31] and the value of 100% which we determined could be simply random.

HIV-1-p24-antigen/anti-HIV 1/2 was ultimately detected with the Abbott ARCHITECT system in the eluted whole blood of all 112 infected persons. Thus, the rather unfavourable routine elution conditions used in this investigation proved to be in no way disadvantageous. The determined ideal analytic specificity and sensitivity of 100% each were not only equal or superior to the performance characteristics established with other immunoassays which have been specifically adapted to DBS testing [38,39] or a stepwise procedure with the combined use of several anti-HIV tests [40]. They, indeed, also exceeded the performance record of an assay that had been specially developed and optimised for the detection of anti-HIV antibodies in DBS eluates (Q-Prevent HIV 1 + 2 DBS kit) [41].

## Conclusions

The performed study shows that it is nowadays possible to reliably detect HBsAg, anti-HCV and HIV-1-p24-antigen/anti HIV 1/2 with state-of-the-art analytical systems such as the Abbott ARCHITECT in DBS eluates even when a comparatively high elution volume of 1,000 μl is used and modifications of the protocols established for serum or plasma investigations are largely dispensed with. However, the communicated results also confirm the inherent analytical limits of DBS testing. It fails, for example, completely in the anti-HBc/anti-HBs system for those individuals infected with HIV due to the low antibody concentrations; it proved to be definitely problematical in the recognition of HCV infections which had long since been resolved; and it required molecular biological procedures with optimal analytical sensitivity with regard to HBV DNA and HCV RNA tests. Due to our findings, the projected screening algorithm for the upcoming cross-sectional study “Drugs and Chronic Infectious Diseases” was modified. Individuals infected with HIV will be always tested for the presence of HBV DNA. Participants whose DBS eluates are positive for anti-HBc or anti-HBs should be subjected to a venipuncture during a second consultation in order to definitely clarify their anti-HBc/antiHBs status and, finally, all DBS eluates will be screened for HCV RNA regardless of the results of anti-HCV testing.

## Methods

Paired serum/DBS samples obtained from 726 patients were included into this study. Upon admission to our hospital, patients provided written consent to all necessary biochemical, bacteriological, and virological investigations. All samples used throughout the study were sent to our laboratory in the process of routine clinical diagnostics. Thus, none of the specimens was collected specifically for the purpose of the study, not a single additional venipuncture was performed and none of the materials was tested for any parameter other than those required by the physicians in the course of the normal diagnostic work-up. Given these circumstances, approval by an ethics committee seemed to be dispensable.

To prepare the DBS, 100 μl of whole blood were initially applied to a Whatman/Schleicher & Schüll (Dassel, Germany) #903 filter paper and subsequently dried overnight [4,6]. For the five antigen and antibody tests the DBS were eluted with 1,000 μl of a buffer (PBS/0.05% Tween 20/0.08% sodium azide) overnight at room temperature on a shaker [16]. This was followed by centrifugation of the eluates for two minutes at 10,000 rpm. The whole blood eluates for the performance of HBV DNA and HCV RNA tests were obtained under identical conditions in a second operation.

**Figure 2:**
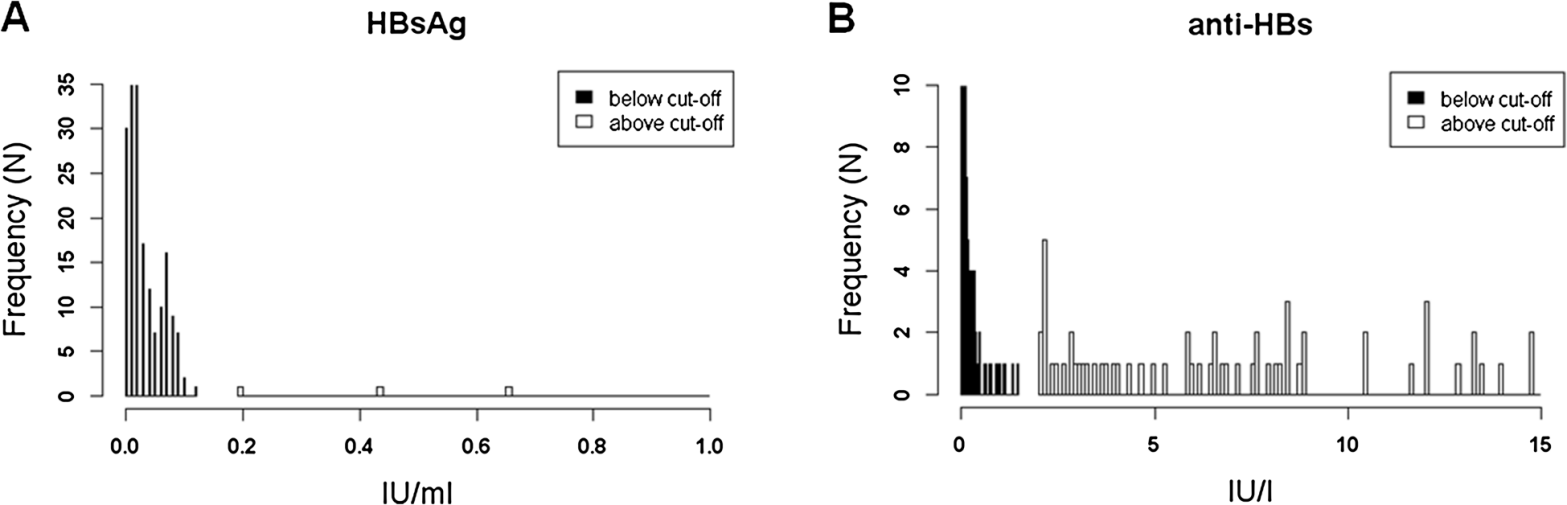
**HBsAg and anti-HBs testing in DBS eluates.** Separation of positive from negative materials after changing the cut-off values to 0.15 IU/ml (HBsAg) **(A)** and 1.5 IU/l (anti-HBs) **(B)**, respectively.

The DBS eluates as well as the corresponding sera were tested for HBsAg [42], anti-HBc [43], anti-HBs [44], anti-HCV [45] as well as for the simultaneous presence of HIV-1-p24-antigen and antibodies against HIV 1/2 (HIV Ag/Ab Combo) [46] using the ARCHITECT system (Abbott Diagnostics, Delkenheim, Germany). The measurements generally adhered to the manufacturer’s recommendations, but had to be modified for two parameters in the course of DBS testing. Due to the unavoidable haemolysis, it was necessary to increase the cut-off value of the HBsAg determination from ≥ 0.05 to ≥ 0.15 IU/ml. On the other hand, to compensate for the effect of dilution the threshold of a positive reaction to anti-HBs was lowered from ≥ 10.0 to ≥ 1.5 IU/l. These changes in the cut-off values neither impaired the discrimination of HBsAg-positive and –negative samples nor affected the assignment of anti-HBs-reactive and –non-reactive materials. However, as shown in Figure 2, the interval separating positives and negatives was consequently lowered to 0.12 - 0.19 IU/ml (HBsAg) and 1.45 – 2.07 IU/l (anti-HBs). Abbott HBsAg neutralisation tests as well as anti-HCV and anti-HIV immunoblots were not used in the context of DBS testing because the tentative protocol of the projected study “Drugs and Chronic Infectious Disease” specified that every reactive HBsAg and anti-HCV finding in the immunoassay is to be verified by a nucleic acid test and that positive anti-HIV results established in DBS eluates are to be confirmed by two independent analyses from serum.

Serum and DBS eluates were subjected to nucleic acid extraction by the MagNa Pure 96 system using Viral NA Universal kit (Roche Diagnostics, Mannheim, Germany). The artus HBV LC PCR (Qiagen, Hilden, Germany) [22] and the VERSANT HCV RNA qualitative (TMA) systems (Siemens Healthcare Diagnostics, Eschborn, Germany) [33] were used for the qualitative determination of HBV DNA and HCV RNA; their detection limits are 100 IU/ml and 5 IU/ml, respectively. The b-DNA technology was employed for quantification in serum (VERSANT HBV bDNA 3.0 assay and VERSANT HCV RNA 3.0 Assay, Siemens Healthcare Diagnostics, Eschborn, Germany) [47-49].

The data was analysed with Predictive Analytics Software (PASW), Version 18 (SPSS, Chicago, IL, USA). Confidence intervals were calculated according to Clopper and Pearson [50] employing the R Project for Statistical Computing (available at: http://R-project.org. Last accessed: 10 March, 2013).

## Competing interests

The authors declare no competing interests.

## Authors’ contributions

RSR, OS, UM, WC, WZ, RZ, and MR designed and conceived the study. Data were collected and provided by RSR, OS, NG, WC, WZ, and RZ and were analysed by RSR, OS, NG, WC, WZ, and RZ. RSR wrote the manuscript that was read and approved by all authors.
